# Conceptualizing Municipal Elections: The Case of Toronto 2018

**DOI:** 10.1177/10780874211031155

**Published:** 2021-08-04

**Authors:** J. Scott Matthews, R. Michael McGregor, Laura B. Stephenson

**Affiliations:** 1 Memorial University, St. John's, Newfoundland and Labrador, Canada; 2 Ryerson University, Toronto, Ontario, Canada; 3 Western University, London, Ontario, Canada

**Keywords:** elections, levels of conceptualization, Toronto

## Abstract

Since Angus Campbell and colleagues first introduced the “levels of conceptualization” (LoC) framework as a measure of political sophistication, a number of scholars have applied the approach to subsequent American national elections. In this study, we present the first application of the LoC framework to a municipal election, and focus upon the 2018 Toronto mayoral race. After describing the method and data we use to adapt the framework to this new context, we replicate previous analyses, and find that LoC is related to local voter turnout and several measures of political sophistication. We then consider the question of whether major candidates were discussed at different LoC, and if their supporters view local politics at different LoC. We conclude by making the case that the LoC framework is helpful for resolving the debate over whether local politics are ideological or managerial in nature.

## Introduction

In the not-too-distant past, it was common for scholars to bemoan the weak state of the literature on municipal elections and voting. Less than a decade ago, Marschall, Shah, and Ruhil (2011) went so far as to state that, “to say that a field of study on local elections exists would be a bit of an overstatement.” Cutler and Matthews (2005) noted, “Municipal elections are the poor cousins in the study of elections and voting behaviour.” In recent years, however, there has been an explosion in the study of these contests, including in Canada, and this has greatly increased our understanding of how Canadians make choices about who governs them (see Lucas and McGregor 2021a, for a detailed account of the recent rapid growth of the subfield). The reasons for this growth are simple: the sheer number of municipal elections, along with the institutional, historical, cultural, and other dimensions of variation they provide, creates a unique opportunity for studying a variety of new and important questions about voting behavior, and researchers are taking advantage of it.

Local elections offer new contexts in which to consider theories and approaches that have been developed for higher order elections, and the unique nature of local contests poses both a challenge and an opportunity to scholars. On the one hand, there is reason to believe that municipal elections are similar in kind to other voting events—they have candidates, issues, and campaigns, so why would voters not approach them in the same way? Canonical models of voting behavior that prioritize a “funnel of causality” (Campbell et al. 1960; Miller and Shanks 1996) have been found useful at the municipal level (McGregor, Moore, and Stephenson 2016; Bélanger et al. 2022), demonstrating that, in important ways, voters in local contests behave in much the same way that they do in national elections.

On the other hand, there are numerous reasons to expect that voters will reason and behave in a unique manner municipally. Research shows that electors are less interested in local than national politics, tend to believe that local government matters less to their lives, and feel less of a “duty” to vote in local elections (McGregor, Moore, and Stephenson 2021). These findings are unsurprising given that municipal turnout rates are substantially below those witnessed in national and provincial elections. Relatedly, participants in local elections are different from those at higher levels—home owners and individuals who have lived in a city for a prolonged period of time, for example, make up much larger shares of the voting population locally than they do in national elections (Dostie-Goulet et al. 2012; Fischel 2001; Nakhaie 2006). Finally, the scope of authority for municipal elections, especially in Canada, is significantly different from that at other levels of government. Not only are the areas of policy responsibility different, but so are the (limited) budgets and taxation capacity. While provincial and federal elections can bring changes to major social programs and foreign policy, municipal elections can lead to changes in recycling programs and zoning priorities.^1^ It is therefore an open question whether municipal policy imperatives can even align on an ideological dimension the same way that policy choices do at other levels.

The purpose of this work is to begin to probe this issue by investigating how voters think about municipal candidates and elections. To that end, we draw upon the levels of conceptualization (LoC) framework introduced by Campbell et al. (1960). With this approach, the open-ended responses of survey participants are categorized according to how candidates or parties are discussed. Categories include ideology, group benefits, as well as more short-term considerations, such as performance and/or issues. Initially developed to assess the sophistication and ideological coherence of voters, we contend that the framework also provides an invaluable way of assessing a debate in the academic literature (introduced in detail below) about whether local politics is ideological or managerial in nature. That is, we consider the question of whether voters think about municipal politics in ideological, structured terms or whether they are prone to concentrate on more proximate considerations related to municipal management.

Since being used in *The American Voter*, scholars, such as, Field and Anderson (1969); Verba, Norman, and Petrocik (1978) and Lewis-Beck et al. (2008) have used the LoC framework to update our understanding of the American public. Though the measure has been disputed as a valid indicator of political sophistication (Smith 1980 but see Cassel 1984), we believe the LoC framework can be an important tool for understanding the lens through which Canadian electors view local elections. Accordingly, this work applies the LoC to the 2018 Toronto mayoral election, utilizing a set of open-ended survey questions that asked respondents to indicate what they liked or disliked about major candidates. We coded responses to correspond with LoC categories, providing insight into the factors that spring to mind when electors are prompted to think about local candidates, and thus local elections.

In important ways, the 2018 Toronto mayoral race was typical of Canadian local elections. The incumbent, John Tory, won handily in a race where the voter turnout rate was just over 40%. Tory, who was first elected in 2014, faced just one credible challenger, former Chief City Planner for the City of Toronto, Jennifer Keesmaat. There were 33 other candidates, but none seriously challenged the front runners (the third-place finisher received just 3.4% of the vote). After leading in the polls for the entirety of the campaign, Tory captured nearly two-thirds of the vote (winning in every ward) and was re-elected by a margin of forty percentage points. These features—a dominant incumbent candidate and middling turnout—are common place in Canadian local elections, making the 2018 Toronto election a good case with which to begin the study of LoC in the local context.

In the following sections, we outline a debate in the literature about the nature of local elections, explain the LoC framework and how it is useful, and discuss how we apply it here. We then present an analysis of data gathered during the 2018 Toronto municipal election period. Our analysis begins with a qualitative analysis of the content in each LoC level and then moves to a quantitative replication of influential analyses from Lewis-Beck et al. (2008). These sections validate our application of the LoC framework and map out the way in which Torontonians viewed their candidates for mayor. We then turn to consider whether the two major candidates were viewed in different LoC terms, and if this relationship is dependent upon vote choice, a novel application of the framework. Our results demonstrate that while the LoC does not provide a robust measure of sophistication, it does reveal that most municipal voters do not think about candidates in ideological terms. However, we do see candidate-specific variation, with supporters of the challenger more likely to reference both candidates in ideological terms. We conclude with a discussion of why such a candidate-specific pattern may exist. On the whole, we find that the LoC framework is a promising tool for understanding local political behavior.

## LoC and Municipal Elections

Though the field is growing, the study of municipal elections is far less developed than that of national contests, which tend to be considered “the” event in electoral democracies. Municipal elections tend to be less engaging, drawing comparatively little attention and fewer voters than is the case at higher levels of government (Kushner and Siegel 2006; McGregor 2018; Nakhaie 2006). One can easily understand why many voters might be drawn to elections that decide matters of foreign policy, national environmental strategy, and the “peace, order, and good government of Canada,” but less intrigued by those who decide garbage pickup, bus schedules, and parking by-laws. It is no wonder that municipal elections tend to be less publicized, less exciting and, in turn, less salient for voters (Cutler and Matthews 2005). Beyond the specific policy domains, municipal elections often diverge from their national cousins in that they often lack the involvement of political parties. In many places in North America, including most Canadian cities, mayoral and city council candidates all run as independents—a distinction that is key to our analysis below, as the absence of parties removes an important heuristic that might prime voters to think in ideological terms.

A debate over the fundamental nature of local elections, or, more specifically, the importance of ideological considerations, exists. On one hand, scholars have argued that ideological considerations are significantly less important in the municipal setting than they are in national (or even provincial/state) contests (see Oliver 2012; Peterson 1981). The general argument is that local elections are more about managerial competence than ideology: as many of the issues that municipalities are responsible for have no obvious ideological implications, voters base their decisions upon their expectations of who will perform best, placing particular emphasis upon the performance of incumbents. In this view, low voter turnout rates allow specific segments of the population (such as homeowners) to have an outsized influence on election outcomes. Most importantly for our purposes, however, is the suggestion that local elections are based upon evaluations of the managerial competence of candidates, rather than ideology.

On the other hand, a number of scholars have found that traditional left–right ideology is a significant driver of local vote choice. Recent studies, including several on Toronto, in particular, have shown that, even in a non-partisan municipal contest, ideology (as measured by standard left-right indicators) is a significant determinant of mayoral vote decisions, and that voters make ideological assessments of candidates (Sances 2018; see McGregor, Moore, and Stephenson 2016; McGregor and Pruysers 2021; and Stephenson, McGregor, and Moore 2018, on Toronto). In reviewing the determinants of vote choice in mayoral elections in eight Canadian cities, Lucas and McGregor (2021b) conclude that ideology is “deeply important at the urban scale,” and argue that there is little evidence that these elections are driven by “non-ideological assessments of managerial performance.” These findings are congruent with the conclusion of Cutler and Matthews that municipal elections are “different in degree but not in kind from the national level” (p. 360).

The existing literature on determinants of vote choice in local elections, and on how local elections differ from those held at other levels, consider many factors which are conceptually independent of the management-ideology distinction. For instance, a noteworthy segment of this literature has argued that these contests are driven largely by sociodemographic cleavages, with qualities, such as, homeownership (Fischel 2001), race (Hajnal and Trounstine 2014), and class (Bridges 1997; Trounstine 2008) being central to determining candidate support. As scholars have noted (Hajnal and Trounstine 2014; Sances 2018), though these factors can be strongly correlated with left–right ideology, they are nevertheless distinct from one another. Studies of Toronto, in particular, have found that vote choice is influenced by a variety of factors, including sociodemographic characteristics, partisanship, issues, economic evaluations, geography, and strategic considerations (Anderson et al. 2017; Caruana et al. 2018; McGregor, Moore and Stephenson 2021; McGregor and Pruysers 2021). Given that both former Mayor Rob Ford, who held office from 2010 to 2014, and his brother Doug, who was a mayoral candidate in 2018, are well known for their anti-establishment positions and styles, the subjects of populism and populist attitudes have been a particular focus of attention in Toronto (Kiss, Perrella, and Spicer 2020; Silver, Taylor, and Calderón-Figueroa (2020)). Many factors other than ideological or managerial considerations are known to have a relationship with mayoral vote choice.

It is not the purpose of the current work, however, to either test or dispute the applicability of these explanations to the 2018 race. Existing findings are not incompatible with contentions that either managerial or ideological concerns drive local politics. Indeed, there may be relationships between any of these factors and the manner in which local politics are conceptualized. The presence of correlations does not speak to the manner in which electors conceptualize local politics. Therefore, despite the fact that most evidence from Canada, including from Toronto in particular, suggests that ideology is fundamental to mayoral elections, we see value in plumbing the ideology-versus-management debate further, from a different angle. Our application of the LoC framework enables us to probe voters’ opinions directly to determine the relative shares of the population who focus on ideological or managerial considerations. The LoC framework, in which respondents are categorized with respect to the type of considerations that are “top of mind,” speaks directly to this debate, categorizing respondents on the basis of open-ended responses.

We are keenly aware, however, that it may be foolhardy to expect ideology to be a major consideration for any voter at any level. The initial development of the LoC framework in *The American Voter* (Campbell et al. 1960) was designed to evaluate the sophistication of the electorate. Respondents in that study were classified in terms of how they conceptualized politics on the basis of their open-ended responses to questions about parties and candidates. They found that relatively few individuals (one in four self-reported voters, or one in five of the total sample) conceptualized ideologically according to their classification scheme, which identified ideologues, near-ideologues, those concerned with group benefits, those who focused on the nature of the times, and those who had no issue content at all (further details on these categories are provided below). Despite disagreements about the typology, from having fewer (Field and Anderson 1969) to more (Nie et al. 1979) categories, and the validity of the measures (Smith 1980, 1981, 1989, but see Cassel 1984 and Hagner and Pierce 1982), the overall finding of weak ideological constraint is well-supported (Converse 1964; Coveyou and Piereson 1977; Field and Anderson 1969; Klingemann 1973, 1979; Nie et al. 1979; Pierce 1970).

However, we believe that the LoC framework can also push our understanding of municipal politics in other ways. Knight (1985) suggests that we can learn more about how voters reason, and what they reason about by taking into account their ideological sophistication. Utilizing the LoC framework, she finds that the impact of party identification and issue preferences on candidate support varies depending on one's ideological coherence. Further, Lewis-Beck et al. (2008) suggest that interpreting electoral results is facilitated by a better understanding of how voters think and reason about politics. Therefore, we believe there is value in using the LoC, as originally formulated by Campbell et al. (1960), as an exploratory framework to not only probe the ideological or managerial nature of municipal elections but also push our understanding of municipal voters. For our purposes, finding almost no ideological content in municipal considerations would suggest a managerial view of municipal elections.

The objective of this paper is to extend the use of LoC to identify the principal ways that people understand—that is, conceptualize—municipal politics, as indicated by statements they make when evaluating candidates. Local electoral competition can be difficult to understand because the elections are not always as obviously ideological or partisan as races fought at higher orders of government. As Lewis-Beck et al. (2008: 257) put it, we seek to know “[w]hat cognitive structures do [voters] bring to bear on parties and candidates? Into what conceptual categories do they place the candidates, parties, and other stimuli that emerge during an election campaign?” The LoC framework can help us do this. We want to know whether municipal voters reason about municipal candidates in abstract, ideological terms; in terms of groups benefits; in terms of proximate events (or “the times”); or nothing at all, and if their reasoning depends upon the candidate being assessed. While the use of the LoC framework is not new, this is the first time it has been used at the municipal level, and the first time it has been applied to a non-partisan, low-interest contest.

## The 2018 Toronto Mayoral Election and the Canadian Municipal Election Study

Our analysis is based upon survey data collected at the time of the 2018 Toronto mayoral election. In many ways, this contest was typical of elections in Canadian cities: the non-partisan race saw an incumbent comfortably re-elected with low voter participation. Though the race included 35 candidates, only two can be described as major contenders: incumbent John Tory and challenger Jennifer Keesmaat. Together, the duo accounted for 87.1% of the vote. The third-place finisher was “alt-right” candidate Faith Goldy, who recorded a mere 3.4% vote share (at no point was the candidacy of Goldy or any other minor candidate considered serious enough for them to be included in commercial polling). Voter turnout was quite low, at about 41%, a significant decrease from 2014 when it was just shy of 55% (City of Toronto).

Mayor Tory was first elected in 2014 with 40.3% of the vote, beating out Doug Ford (33.7%), brother of former Mayor Rob Ford, and Olivia Chow (23.2%), a former NDP Member of Parliament and widow of the former leader of the party, Jack Layton. Prior to his election, Tory served as a business executive and as leader of the provincial Progressive Conservative Party from 2004–2009. Keesmaat's most high profile previous position was as chief city planner of Toronto, a role in which she served from 2012 to 2017. Keesmaat entered the mayoral contest on the nomination deadline, meaning that her campaign period was much shorter than the mayor’s campaign period. The 2018 election was far less exciting than the 2014 contest. Tory maintained a sizable and steady lead in opinion polls throughout the campaign and won easily, with 63.5% versus Keesmaat’s 23.6% vote share. While any election in Toronto garners attention because of the size, importance, and budget of the city, the 2018 mayoral campaign was largely uneventful.^2^

Every election is to some degree unique, and there is always a need to be careful about generalizing from a single case. Nonetheless, we argue that the 2018 Toronto election can provide a useful first cut at adjudicating between the relative importance of ideological and managerial considerations at the local level. Given the presence of a popular incumbent on the ballot, we might expect managerial considerations to dominate. Both major candidates had a managerial “record” on which to run: voters were able to judge Tory's managerial competence on the basis of his mayoral record and, for her part, Keesmaat was most well known for her experience as Chief City Planner, a high profile position which could signal a high degree of managerial competence even if one did not know the details of her record. At the same time, the two frontrunners were widely seen as ideologically dissimilar. Our survey (which is described in further detail below) asked respondents to position the candidates on a left–right (0–10) scale. Tory received an average score of 6.26 and Keesmaat received 3.78—the difference of 2.48 points is significant at *p* < .001.

Our analysis is based upon data from the Canadian Municipal Election Study (CMES), which includes large-N surveys of electors in eight cities. The study included pre- and post-election survey waves with questions similar to those asked in national-level election studies in many countries. As part of the CMES, respondents in Toronto were asked what they liked and disliked about the two major candidates. Respondents were also asked a series of standard questions about attitudes, behaviors, and motivations relevant to the election. In total, 2,400 respondents completed the pre-election survey, with just shy of 1,600 also completing the post-election questionnaire.^3^ Surveys were conducted online with recruitment conducted via phone and from online panels.^4^

## Methodology

Respondents were asked to express their likes and dislikes regarding the candidates using open-ended text boxes (see complete details in Appendix I of the Supplemental Material). Respondents were first asked what they liked about each candidate, and then a parallel battery followed asking what they disliked about each candidate. Each item also allowed two closed-ended responses: “There is nothing I like [dislike] about this candidate” and “Don't know.”^5^

To categorize these responses according to the LoC framework we designed an original coding protocol based upon discussions in Campbell et al. (1960) and Lewis-Beck et al. (2008). Consistent with past research, our LoC coding consists of five levels (discussed in more detail in the next section):

*A1: Ideology and issues*. References to ideological concepts or a “broad abstract judgmental standard” (Lewis-Beck et al. 2008, 261) that are clearly connected to “issues,” whether policy controversies or performance considerations.
*A2: Ideology, not issues*. References to ideology or abstract standards that are *not* explicitly connected to issues.
*B: Group benefits*. Associations between the candidates and perceived benefits (or costs) they are expected to deliver to (or impose upon) particular social groups.
*C: Performance and/or issues*. Associations between the candidates and past performance, or expected future performance based on past experience, either generally or with respect to specific issues or issue themes.
*D: No issue or performance content*. Valid responses that contain no identifiable issue or performance content.

Trained coders assigned each non-blank, open-ended response to one of these five categories or, for those responses that were non-blank but reflected a form of non-response (e.g., a non-sensical string of characters), to the category “NR: Non-response.” We then combined the response-level coding (i.e., the codes assigned to each like or dislike response) to assign an overall LoC to each respondent. Respondents were assigned to the “highest” level (where A1 > A2 > B > C>D) observed across their four responses. Statistical measures of the reliability of the resulting measure exceed the standard threshold for “tentative conclusions” (Krippendorff, 2004). Further, differences between respondents in the ideological categories (i.e., those coded to either A1 or A2, taken together) and the other levels are especially reliably measured. Complete details of our reliability study are reported in Appendix II of the Supplemental Material.

While our measure of LoC is faithful to past usage, we note certain important differences between our approach and earlier work. Perhaps most significant is the mode difference: whereas the CMES data were collected online, prior work using the American National Election Study (ANES) relied on face-to-face interviewing and verbatim transcription of responses. The high level of the interviewer–respondent rapport that can be established in a face-to-face setting seems likely, on its own, to motivate more elaborate and possibly more considered responses than in an online setting. In addition, the ANES queries likes and dislikes in relation to parties as well as candidates. Further, the items used in Campbell et al. (1960) included follow-up probes after each response, and the authors report that this part of the interview might have lasted as long as 15 min (p. 222, fn. 3). Altogether, these differences suggest that our open-ended responses are very likely to be less extensive, which almost certainly reduces the reliability of the coding relative to what it would be with more extensive materials. For the same reason, however, our measure may be more valid than those used in earlier works, inasmuch as ANES face-to-face survey procedures provoke a level of cognitive elaboration about politics that is not representative of “everyday” thinking about politics. We note also that the ANES’ queries about party evaluations, which would be impossible in the non-partisan context of Toronto politics, may evoke ideological or group-centered associations to a greater degree than the strictly candidate-focused items on which our analysis relies.

Important for our analysis of the debate over whether local politics is ideological or managerial, some of the categories can be interpreted as evidence in one direction or the other. The first two categories (A1 and A2) are obviously indicative of ideological thinking and would provide support for the ideology side of the debate if a large share of the population is coded as such. Category C (performance and/or issues) suggests a “management” approach, as it includes performance evaluations (either past or future). If a large share of the population evaluated the candidates at this level, the managerial side of the debate would be supported. Categories B (group benefits) and D (no issue or performance content) are not obviously related to either category.

## LoC in Toronto: Illustrative Examples

In this section, we provide a qualitative description of the LoC as manifested in our Toronto data. We selected illustrative examples of each category by, first, drawing a random sample of ten respondents from within each category and, second, selecting from the sample a set of examples that jointly capture the range of variation of the responses within that set. Our goal is to provide a sense of the responses that is roughly representative of our data. (Note that we have not edited the responses. Truncated responses end with an ellipsis.)

*A1: Ideology and issues.* Respondents in this category must refer to ideological concepts or other abstract standards and rely on them explicitly in characterizing and evaluating the electoral environment, especially in relation to issue controversies and performance judgments. Campbell et al. (1960) described these individuals as “ideologues.” Yet, as in *The American Voter,* our respondents are hardly armchair political philosophers. Connections between ideological categories and specific issues are often somewhat elliptical. The responses vary considerably, furthermore, in their elaborateness and degree of polish. Importantly, as in Campbell et al. (1960) and Lewis-Beck et al. (2008), neither objective accuracy nor political sophistication is required to be placed in category A1, though as we will see in subsequent sections of this paper, there is a correlation between the latter and the ordinal LoC indicator.

One particularly well-expressed and even-handed response made reference to both the abstract goal of “fiscal responsibility” and the “progressive” ideological label:

[*Anything in particular you like about John Tory?*] He is fiscally responsible by not over spending on items that have little benefit to the largest percentage of people.

[*Like about Jennifer Keesmaat?*] Her planning platform is progressive and would encourage modern transit and development befitting a world-class city.

[*Dislike about Tory?*] He can be too willing to accommodate everyone’s opinion rather than following his own vision.

[*Dislike about Keesmaat?*] Her plan is not fiscally sound nor does it identify realistic funding sources.

The set of responses reveals a highly differentiated view of politics that, in addition to raising ideological concepts that are directly connected to distinct policy issues, also points to questions of political leadership (i.e., the reference to Tory’s readiness to follow “his own vision”).

A more typical “ideology and issues” response may be the following:

[*Like about Tory?*] Moderation, integrity, fiscal responsibility

[*Like about Keesmaat?*] Creativity, progressive politics, housing policy

[*Dislike about Tory?*] Scarborough subway policies

[*Dislike about Keesmaat?*] A bit too liberal

Notably, like the first example, this response raises a variety of ideological concepts and abstract standards “too liberal,” “progressive,” along with both policy issues (Scarborough subway policies) and leadership qualities (integrity). Yet connections between ideology and issues are less explicit and must be inferred from the close coincidence of the two types of references. The response is essentially a point-form listing of pros and cons, an approach that may have been suggested by our question’s request for “up to three” likes/dislikes.

A final example reflects a respondent with strong negative reactions to both candidates; indeed, this respondent declined to express anything they liked about either Tory or Keesmaat. Like the other responses presented in this section, the example also refers to both policy and leadership considerations:

[*Dislike about Tory?*] He is weak. Has no backbone, and must test the winds before making a decision. People pleaser.

[*Dislike about Keesmaat?*] She is Marxist in her wealth redistribution ideas. The last idea (taking some $ from $4M home owners to finance affordable housing,) does not make mathematical sense.

*A2: Ideology, not issues.* Respondents in this category are “near-ideologues” in Campbell et al.’s (1960) parlance: they refer to ideological concepts or other abstract standards, but direct connections to policy or performance considerations are not necessary. While these respondents sometimes refer, across their separate open-ended responses, to both ideology and issues, the issue references are not directly connected to ideological or other political abstractions. What separates A1 from A2 is clear evidence of integration between ideology/abstract standards and specific features of the electoral landscape.

One group of near-ideologues provided point-form-type reflections on the candidates that combined isolated ideological mentions with references to issues or personal and leadership qualities (each group of responses, below, was provided by a different respondent):

[*Like about Tory?*] centrist

[*Like about Keesmaat?*] focus on transit^6^

[*Like about Tory?*] Can work with people know how to run a city

[*Dislike about Keesmaat?*] to far left

[*Like about Tory?*] Moderation

[*Like about Keesmaat?*] Passionate, forward-thinking, well-informed

[*Dislike about Tory?*] Cautious, parochial, opportunistic

[*Dislike about Keesmaat?*] Dogmatic

[*Like about Tory?*] Civility, bipartisanship, articulateness

[*Like about Keesmaat?*] Kind of cute

[*Dislike about Tory?*] Dithers

[*Dislike about Keesmaat?*] One of cute

While brief, some of the responses convey a familiarity with political concepts beyond ideology (e.g., opportunism, bipartisanship), which may suggest a high level of political sophistication. Contrarily, one response suggests a certain ambivalence or confusion about the respondent’s underlying preferences (e.g., disliking both Tory’s opportunism *and* Keesmaat’s dogmatism).

Other respondents were more elaborate:

[*Like about Tory?*] Willing to look at other ideas. Tries to bring people together Defends the city’s interests against other levels of government

[*Like about Keesmaat?*] Has an inspiring vision of what the city could be like in the future Not particularly politically affiliated Advocates for making the city more sustainable and environmentally responsible

[*Dislike about Tory?*] May be unduly influenced by the Conservative party.

This response is as extensive and well expressed as any in the A1 category. Importantly, the reference to abstract standards (i.e., environmental responsibility) is not explicitly linked to a particular issue.

*B: Group benefits.* Respondents in this category saw the candidates in terms of their perceived affinities with various social groups, particularly the costs/benefits they were likely to impose on or provide to those groups. Some respondents mentioned associations with multiple groups. Respondents also often mentioned particular issues of concern and sometimes these were explicitly related to the social group(s) in question.

A large subset of these respondents referred to economic interests of one kind or another. In addition to identifying Keesmaat with gender equality, one respondent understood Tory principally with reference to social class:

[*Like about Keesmaat?*] She is committed to LRTs instead of overly expensive and unnecessary short subway lines in the suburbs. She is committed to gender parity. She has never urged Kim Campbell to mock a man’s face because of the lasting, visible effects of Bell’s …

[*Dislike about Tory?*] He hasn't done enough to stand up to Doug Ford. He is too rich to understand what living in the city is like for most working and poor people. He seems to be okay with the racist police practice of carding. He has promoted some of the worst …

[*Dislike about Keesmaat?*] She wants to lower the speed limit to an unreasonably low 30 km/h. … and I say this as a father with two kids under 10 who play outside and walk to school.

A notable feature of the response is the salience of not only class but of *class conflict* (i.e., as a rich person Tory cannot “understand” the concerns of “working and poor people”). Such an explicit reference to competition between groups is a more elaborate manifestation of group-centered thinking than found in many other responses mentioning group interests. See, for example, the following example concerning an economic group (people living in poverty):

[*Like about Tory?*] He is very down to earth and a good listener to people. He is trying to create positive changes in the city.

[*Dislike about Tory?*] He has to do more for people living in poverty.

As regards non-economic group references, none in the sample examined explicitly invokes group competition. That being said, the following example, which refers to immigrants, presumably rests on a perceived, implicit competition of interests between immigrants and non-immigrants:

[*Like about Tory?*] Experienced politician

[*Like about Keesmaat?*] Planning abilities since she was the city planner

[*Dislike about Tory?*] Bends over backward to cater to immigrants, even as Toronto is overwhelmed by immigration, more than any Canadian city.

[*Dislike about Keesmaat?*] She seems to be making expensive promises that will cause a massive property tax increase.

*C: Performance and/or issues.* Respondents in this category refer to associations between the candidates and “good times and favourable conditions” (Lewis-Beck et al. 2008, 272) or to their opposite. References may be to past governing performance or to expected performance based on past experience and may be specific to a particular issue or theme, a set of issues, or highly general. The latter type of reference, an association between a candidate and a highly diffuse performance evaluation, is what typified the category for Campbell et al. (1960), who labeled these as “nature of the times” references. One important restriction on the category is that the reference must be to associations with “external environmental conditions” (Lewis-Beck et al. 2008, 272)—that is, to social, economic, or other performance outcomes that are the perceived result of government action—rather than to political or administrative competence or to improper conduct. For instance, references to political scandals, as such, do not fall within category C.

One respondent combined positive and negative performance judgments of Tory with largely character-centered reflections on Keesmaat:

[*Like about Tory?*] Realistic approach to spending

[*Like about Keesmaat?*] She is high profile enough to keep Tory from being complacent.

[*Dislike about Tory?*] Needs to be more forceful, less political when dealing with provincial and federal governments.

[*Dislike about Keesmaat?*] Unrealistic ideas. Annoying.

A second respondent made quite similar remarks, combining a divided performance assessment of Tory with character-oriented comments on Keesmaat:

[*Like about Tory?*] has regular news updates, has been very visible in what he does, personable

[*Like about Keesmaat?*] like that she is a professional city planner, has new ideas and is a woman.

[*Dislike about Tory?*] doesn’t seem to take a stand, i.e. tries for consensus when he needed to stand up to Doug Ford.

[*Dislike about Keesmaat?*] don’t know yet!

One contrast between the immediately above and previous respondents is that the former is mostly free of references to concrete policy controversies.

Other respondents in this category simply referred to a particular issue—that is, policy concern—without offering any reflections on performance:

[*Like about Keesmaat?*] I like her plan for affordable housing, unlocking the golf courses that are hardly being used for more land and her attitude

*D: No issue or performance content*. Respondents in this category did not mention specific issues or reflect on past performance; rather, the focus of the responses is almost entirely on the candidates’ character or competence. Interestingly, none of the responses examined for the present analysis contained a complete sentence, though, of course, such responses may exist in the larger data set. Notably, the sample of ten responses is dominated by respondents who focused only on positive features of the incumbent.

A few respondents offered only a single, brief reflection on the incumbent (each response, below, was provided by a different respondent):

[*Like about Tory?*] Like him so much

[*Like about Tory?*] His experience

[*Like about Tory?*] Brought an even hand to office

Others who focused only on their positive view of the incumbent were just slightly more expansive:

[*Like about Tory?*] Manner of speaking, calm attitude,

[*Like about Tory?*] Sincere, wellspoken, knowledgeable

Of those respondents who covered both candidates, and included both positive and negative evaluations, the following is typical

[*Like about Tory?*] experience, practical

[*Like about Keesmaat?*] intelligent, new ideas

[*Dislike about Tory?*] complacent

[*Dislike about Keesmaat?*] argumentative

Given our description of the categories and our examples of responses, it should be clear that our respondents had quite varied impressions of the 2018 Toronto mayoral candidates. This provides important variation for our quantitative LoC analyses, reported in the section below.

## Results

We present our quantitative analysis in two stages. First, we replicate the analysis conducted by Lewis-Beck et al. (2008), who themselves replicated the original work from *The American Voter*, to describe the distribution and correlates of LoC among the population. In doing so, we demonstrate the validity of applying the LoC framework to local elections. Note that while we are not focused on measuring ideology to determine the sophistication of the electorate, these analyses follow the initial application of the framework to probe that issue. Second, we conduct new analyses to utilize the LoC framework to better understand voters at the municipal level, beyond the ideological content of their reasoning. In particular, we consider how one’s LoC is related to support for the incumbent and challenger in a low-information, non-partisan context.

### Distribution and Correlates of LoC

We begin by providing an empirical description of our summary LoC measure, as described above. Following Lewis-Beck et al. (2008), we report LoC results for the entire pool of respondents and for voters, separately. These analyses serve the purpose of describing for us how exactly Torontonians viewed the mayoral candidates, which speaks to our primary research question. Table 1 shows the share of respondents in each category, including those who responded “don't know” to these questions (a feature which is very uncommon in other work on LoC).

**Table 1.** Distribution Across the Levels of Conceptualization, by Turnout.

	Entire sample	Voters	Non-voters
A1: Ideology and issues	5.6%	7.2%	1.8%
A2: Ideology, not issues	11.8%	12.6%	9.5%
B: Group benefits	3.2%	3.5%	1.8%
C: Performance and/or issues	53.2%	56.6%	52.7%
D: No issue or performance content	15.6%	13.8%	17.2%
Don’t know	10.6%	6.6%	17.2%
*N*	2209	1,313	169

*Note:* The combined sample size for the analyses of voters and non-voters is smaller because turnout is measured in the post-election wave, which has a lower response rate than the pre-election wave.

The results in Table 1 suggest that explicit ideological thinking is not widespread among Toronto’s electorate. Less than 20% of respondents (even among the subset of respondents who voted) made ideological references when assessing the candidates. These findings are similar to Lewis-Beck et al.’s (2008) analysis of the 2000 ANES data—they found that <20% of the entire sample, and 25% of voters, gave ideological or near-ideological answers when asked about presidential candidates. Though we are somewhat hesitant to make too much of the comparison between ANES and CMES data given the differences in survey mode and questions discussed above, this apparent consistency is nevertheless noteworthy.

The majority of respondents in Table 1 provided assessments based upon performance and/or issues. Recall that it is this category that most closely corresponds with the “managerial” view of local elections. That this category was the highest “level” achieved by a majority of respondents is not surprising given the non-partisan nature of the election. (For comparison, Lewis-Beck et al., 2008 find higher proportions of respondents in the “group benefits” and “no issue content” categories.) Absent of the informational cues of party labels about where candidates might stand ideologically and with respect to group benefits, most voters evaluate the candidates in light of short-term information.

Another striking finding from the table is that a remarkably small number of respondents fall into the group benefits category—just 3.2% of the overall sample. This category is, in fact, the largest in Lewis-Beck et al.’s (2008) sample, at 28.2%. For Torontonians, the mayoral election was very much *not* about group benefits.

In summary, the first column of Table 1 says a great deal about the ideology versus management debate. On balance, the managerial approach seems dominant among our respondents, with many more Torontonians viewing their candidates in managerial than ideological terms. At the same time, a sizable portion of the electorate did view the candidates in ideological terms. Both ideology and managerial considerations are relevant, but to a different degree for different electors.^7^

This conclusion remains unchanged when we focus upon voters alone. At the same time, the comparison of voters to non-voters shows, unsurprisingly, that electors who choose to participate think in more sophisticated terms about candidates than abstainers (a chi-squared test reveals that the difference between these groups is significant at *p* < .001). In particular, voters were considerably more likely to be categorized as “ideologues,” and they were much less likely to provide no assessments of the candidates. This trend is compatible with the findings of Lewis-Beck et al. (2008). When we focus upon voters alone, therefore, the ideology/management balance shifts only slightly.

Next, we consider correlates of the LoC. Although our main focus is not on assessing levels of sophistication, we follow the example of Lewis-Beck et al. (2008) and consider the bivariate relationships between the LoC and three measures that are typically associated with political sophistication: (a) education, (b) political knowledge, and (c) involvement. Education is operationalized by comparing those with and without a university degree. Knowledge is based upon an index of standard political knowledge questions (respondents are categorized as being above or below the median). The measure of involvement, following Lewis-Beck et al. (2008) is based upon an index created from two questions about (a) how much attention respondents paid to the mayoral election and (b) how much of an impact respondents believe local politics has upon their lives (again, cases are divided at the median). Note that the full text of all survey questions employed here can be found in Appendix III of the Supplemental Material. Table 2 shows the results of these three sets of cross-tabulations.^8^

**Table 2.** Levels of Conceptualization and Measures of Political Sophistication.

	Education		Knowledge		Involvement	
	Low	High	Low	High	Low	High
A1: Ideology and issues	3.5%	8.3%	4.3%	7.9%	6.1%	7.2%
A2: Ideology, not issues	9.8%	13.7%	10.7%	13.3%	9.8%	14.7%
B: Group benefits	4.1%	2.8%	3.3%	3.2%	3.2%	3.3%
C: Performance and/or issues	57.1%	56.5%	56.9%	56.6%	55.7%	57.6%
D: No issue or performance content	16.9%	12.6%	14.0%	14.1%	15.0%	13.2%
Don’t know	8.7%	6.2%	10.9%	5.0%	10.3%	4.2%
*N*	492	969	515	946	693	768

Several clear trends emerge from Table 2. First, individuals in each “high” category of political sophistication tend to think at a “higher” LoC than their contemporaries in the “low” category—chi-squared tests reveal that all three sets of relationships are significant at the 99% confidence level. In particular, highly sophisticated electors were more likely to view the mayoral candidates in ideological terms. The second trend that holds across all comparisons is that respondents in the low categories are also more likely to have no opinion of the candidates. All three of the measures of sophistication are related to our LoC categorization in a manner that one would expect.

At the same time, however, the strength of these relationships is very modest. There are many respondents with low levels of education, knowledge, or involvement that view the candidates in ideological terms. The distinctions in the “bottom” categories of the LoC scale are similarly, if not more, modest; there is very little difference, along any dimension, in the share of respondents in categories C or D.

While none of the findings in Tables 1 and 2 is particularly surprising, they do accomplish two goals. First, they provide the first replication of previous work, and application of the LoC framework, in a local election. The distribution of the LoC summary variable reveals significant variation in how municipal voters think about politics. While both ideological and managerial considerations matter, far fewer electors are categorized as ideologues. Second, our results suggest that, as many have argued, LoC should not be taken as a measure of sophistication alone. If one accepts that education, knowledge, and involvement are valid indicators of sophistication, the weakness of the relationships between these factors and LoC suggests strongly that there is much more to LoC than sophistication. We turn now to consider other possible correlates of LoC: vote choice and candidate-specific evaluations.

### Candidate-specific Differences in LoC

We now consider whether there are candidate-specific differences in LoC to see if such an analysis provides additional insight into the manner in which electors view local politics. The stability of LoC has been studied across time (Pierce and Hagnar 1982; Wyckoff 1987). To our knowledge, candidate-specific evaluations have yet to be considered. Given that scholars have tended to conclude that LoC is only marginally reliable across time (Cassel 1984; Knight 1985; Luskin 1987), we suspect that there may also be variation between candidates. More specifically, given the presence of an incumbent (Tory) and the absence of formal partisan cues, we expect there to be a gap with respect to levels of knowledge of the two candidates, and that this gap may lead electors to assess the candidates on the basis of different types of considerations. At the same time, we wish to explore whether supporters of each of the candidates view politics at different LoC. Though we have no clear expectation in this respect, such an analysis could provide additional insight into how electors view local politics and politicians.

We begin by considering the differences between evaluations of Tory and Keesmaat. As noted above, our “overall” LoC measure is calculated by pooling responses to questions about both candidates. We disaggregate responses here to consider the extent to which electors viewed the candidates in similar or different terms. We therefore have two separate LoC measures, based upon the like and dislike questions for each candidate.^9^ We provide the frequency distributions for these measures in Table 3. We show the results both including and excluding “don't know” responses, to determine if the distribution of responses varies if we focus only upon those who provided answers.

**Table 3.** Levels of Conceptualization by Candidate.

	All respondents		“Don't knows” dropped	
	Tory	Keesmaat	Tory	Keesmaat
A1: Ideology and issues	2.8%	2.7%	3.4%	4.1%
A2: Ideology, not issues	7.2%	5.7%	8.9%	8.6%
B: Group benefits	2.1%	1.2%	2.5%	1.8%
C: Performance and/or issues	50.1%	37.5%	61.8%	55.9%
D: No issue or performance content	18.8%	20.0%	23.4%	29.8%
Don’t know	19.2%	32.8%		
*N*	2,385		1,931	1,603

The first two results columns show that respondents were considerably less likely to give answers to the LoC questions when prompted to evaluate Keesmaat. While less than one in five respondents (19.1%) fell into the don't know category when asked about Tory, this value increases to 32.8% for Keesmaat—this difference is significant at *p* < .01. This is no doubt related to Tory's incumbent status, as Torontonians can reasonably be expected to know much more about him than a lower profile challenger.

Among those who did provide a response to these questions, however, did participants view the candidates in similar terms? The answer is, more or less, “yes.” Respondents were slightly more likely to speak of Tory in terms of performance and/or issues (category C), and less likely to fall into the no issue or performance categories (D). This is unsurprising given that Tory’s time in office had provided him with a record to emphasize. There is no discernible difference in the rate at which the candidates are viewed in ideological terms (categories A1 and A2) or in terms of group benefits (B). Though the difference in the overall distributions here are statistically significant (at *p* < .01), these differences should not be overstated (particularly since the differences we see, in categories C and D, are between adjacent categories).^10^

**Figure 1.**
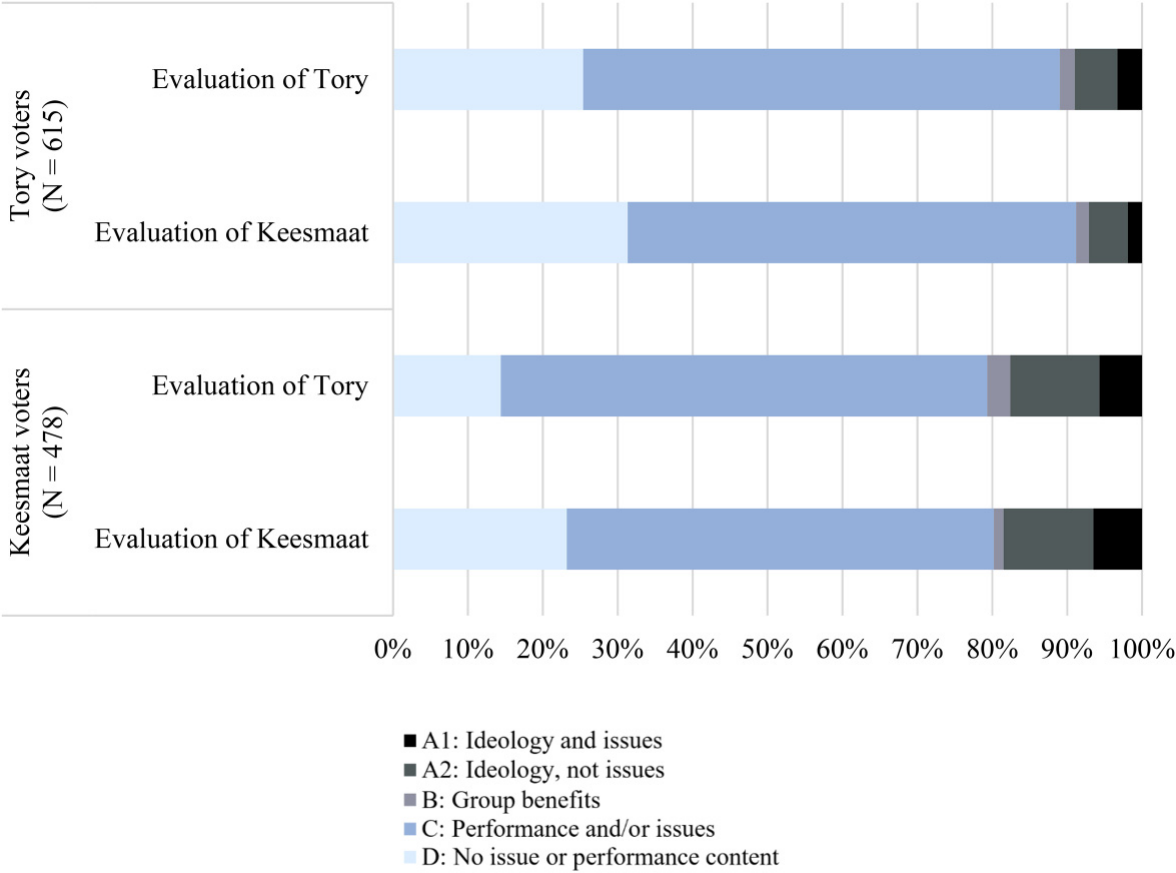
LoC by candidate and vote choice.

In the aggregate, then, among those who do give LoC responses, the views of the candidates tend to be at a similar LoC. Might it be the case, however, that Tory and Keesmaat supporters view the candidates in different ways? It is possible that the candidates attract individuals who focus on different factors when making their vote choices, or that candidates prime their supporters to think at a different LoC. To test this, we present Figure 1, which shows the distribution of LoC according to reported vote choice.^11^ That is, we consider evaluations of Tory and Keesmaat for each type of voter, to see if supporters of the two candidates perceive the two differently.

Two patterns emerge from Figure 1. The less exciting of the two is that, when controlling for vote choice, the differences in perceived LoC of the two candidates remain minimal. The distribution of LoC among Tory voters is similar for both candidates, and the same can be said about Keesmaat voters. In both instances, there are some differences between the sizes of categories C and D (with Tory supporters more likely to be assigned to the performance and/or issues category in both groups), as was the case with Table 3, but there is little to suggest that either set of voters conceptualize the two candidates very differently.

Much more striking is the finding that Keesmaat voters differ significantly from Tory supporters. Keesmaat voters are twice as likely to view *both* candidates in ideological terms (A1 or A2) (*p* < .01 when comparing assessments of both Tory and Keesmaat on the basis of vote choice).^12^ There is no difference, however, in the rate at which candidates are assigned to category C (the category that shrinks among Keesmaat voters is D). These findings suggest that (a) Keesmaat voters are more likely to think of the candidates in ideological terms, but (b) the two sets of voters are equally likely to perceive of the candidates in managerial terms (and such responses remain the most common in all instances). Figure 1 therefore reveals an important finding obscured in Table 3: supporters of the two major mayoral candidates evaluated both candidates in notably different ways.

## Conclusion

Since introduced by Campbell et al. (1960), the LoC framework has been used, modified, adapted, and critiqued by a number of scholars. Until now, however, it has not been applied to the municipal level of politics. Such an omission is noteworthy as the unique nature of local elections means that results obtained from other settings cannot be assumed to travel there. Moreover, given that scholars debate the question of whether local elections are ideological or managerial in nature (Fischel 2001; Lucas and McGregor 2021a; McGregor, Moore, and Stephenson 2016; Oliver 2012; Peterson 1981), it is important to consider how voters view the candidates and, by extension, the choice they must make on election day.

Our detailed analysis of CMES Toronto data reveals several findings of note. First, by replicating previous work on the subject, we have shown that LoC at the local level exhibits many of the same relationships to other variables that have been observed at the national level in the United States. LoC is related to voter turnout, and several measures of voter sophistication, even if the relationship with the latter variables is not tremendously strong. Though not groundbreaking, such replications are nevertheless noteworthy, as they serve to validate our application of the LoC framework at the local level, particularly since there has been no work on the subject either federally or provincially in this country.

More importantly, our application of the LoC framework allows us to speak to the debate over the nature of local political competition. We find that only a minority of voters referred to ideology when asked to evaluate the 2018 Toronto mayoral candidates. Instead, a majority focused upon performance and/or issues. In the aggregate, such a finding is seemingly compatible with the managerial (as compared to the ideological) view of local politics. The fact that a not-insignificant share of respondents did view the candidates in ideological terms, however, suggests that there is noteworthy heterogeneity among the population in this respect. Indeed, given the presence of a popular incumbent on the ballot, which might incline voter thinking toward managerial and performance-centered considerations, our estimate of the level of ideological thinking may be near the lower bound for local elections. Further, we note that the share of Toronto voters who referred to ideological concepts in their reflections on the candidates compares very well with estimates for U.S. presidential elections, which are much more consistently competitive and ideological in nature.

Considering evaluations of the candidates separately sheds additional light on LoC at the local level. Fewer electors were able to express opinions when asked about the challenger compared to the mayor. This is most likely due to different public profiles and the non-partisan nature of the Toronto election. Voters in non-partisan elections lack the informational heuristics that party labels provide, including cues about the ideology of candidates (which can be inferred from past performances by other candidates under the same party banner). Absent partisan and past performance information, electors cannot be expected to know as much about new (challenger) candidates.

Among Torontonians who were able to respond to questions about the candidates, there were only minimal differences in LoC; similar shares of respondents evaluated the two candidates at each of the LoCs. This changes, however, when we account for vote choice. Keesmaat voters were more likely than their Tory counterparts to make ideological references, and they did so regarding both candidates. While “managerial” considerations (category C) were still the most common types of assessments provided, there is therefore a difference in the balance of ideology versus managerial evaluations by the preferred candidate. Keesmaat voters were twice as likely to be ideological in their assessments.

We think this is perhaps the most promising contribution of this study, as it suggests that the future application of the LoC framework can be an invaluable way to better understand the nature of local elections, and is relevant for a variety of subjects, ranging from campaigns, the media, and the personal characteristics of candidates, to the preferences of voters. The question this finding elicits is whether the two candidates attracted voters who thought about politics differently, or if the candidates prompted their supporters to think in different terms. On the one hand, it is plausible that individuals for whom management is important were attracted to Tory, while Keesmaat drew more support from residents that focused on ideology. On the other hand, it is entirely plausible that the two very different candidates that contested the Toronto race influenced the factors that voters considered. Did Keesmaat speak in more ideological terms than Tory, thus convincing her supporters of the importance of this type of consideration? Did the media, for whatever reason, speak of Keesmaat in ideological terms? Do factors, such as candidate gender, incumbency status, charisma, or other personal characteristics prime voters to think in different ways? The truth may be a combination of these possibilities. We expect that LoC may inform political behavior but that it is also informed by political choices. The best way to answer this question, and to learn more about LoC and local politics more generally, is to apply this approach to different settings, perhaps with different numbers and constellations of candidates, higher or lower profile elections, or other features that might affect how politics and politicians are thought of by the electorate. Though our results bring up as many questions as they answer, one thing that is clear is that the LoC framework offers an excellent opportunity to understand how electors view local politics, as well as to probe the effects that specific candidates have upon how the public views elections.

## Notes

1. In Canada, municipal governments have no independent constitutional standing and their rights and responsibilities are subject to the preferences of their provinces.

2. While the mayoral election was largely uneventful, the same cannot be said of the council races. Shortly before the election, the provincial government unilaterally imposed ward redistricting upon the city, reducing the number of council races from 47 to 25, through the *Better Local Government Act*.

3. The pre-election survey was in the field from September 24 to October 21, 2018. The post-election survey was fielded from October 23 to November 22.

4. Forum Research Inc. was responsible for recruitment and administration.

5. No limitation on response length was indicated to respondents, although some responses are truncated in our data set.

6. This response exemplifies a type of borderline case that lies very near the conceptual boundary between levels A1 and A2. It contains both a clear ideological reference (centrist) and an issue reference (transit). Unlike similar point-form-style references coded to level A1, however, the two references do not occur within the same open-ended response (i.e., in response to the same like/dislike question). As such, the level of integration between ideology and issues is less obvious.

7. These conclusions are robust to an alternative analytical approach. In particular, we report in Appendix IV (Table A1) an analysis of the response-level (or question-level) coding, as opposed to the respondent-level coding that is reported in the main text. While this alternative coding is not as reliable as the respondent-level coding (which is unsurprising, given that the latter utilizes more information), it can provide a suggestive indication of the overall mix of considerations mentioned by respondents, whereas the respondent-level coding is designed to identify the highest level of conceptualization indicated by the respondent. As Table A2 indicates, the only notable difference is the higher percentage of respondents included in level D. Many of these respondents will have referred to performance, issue or ideological concepts while, at the same time, mentioning other considerations, such as a candidate's personal characteristics.

8. The reader will note that the sample size for Table 2 is smaller than for Table 1. Table 2 is limited to only those respondents who answered all of the questions necessary to operationalize the concepts included in the table. This group has a modestly higher average level of conceptualization than does the sample from Table 1.

9. Respondents who provided like and dislike answers at different LoC were once again assigned the “highest” value.

10. These conclusions are robust to the alternative analytical approach described in endnote 7. See Appendix IV, Table A1.

11. We present these results in table format in Appendix V, Table A2.

12. This claim holds at both the aggregate and the individual level: an individual-level analysis shows that Keesmaat voters are more likely to view both candidates in ideological terms (5.5%) than Tory voters (2.0%).
